# Effects of transcranial direct current stimulation on patients with disorders of consciousness after traumatic brain injury: study protocol for a randomized, double-blind controlled trial

**DOI:** 10.1186/s13063-019-3680-1

**Published:** 2019-10-17

**Authors:** Shilin Li, Xiangli Dong, Weiming Sun, Na Zhao, Guohua Yu, Lang Shuai

**Affiliations:** 1 Department of Rehabilitation Medicine, The First Affiliated Hospital of Nanchang University, Nanchang, Jiangxi Province 330006 People’s Republic of China; 2 First Clinical Medical School, Nanchang University, Nanchang, Jiangxi Province 330006 People’s Republic of China; 3 Department of Psychosomatic Medicine, The Second Affiliated Hospital of Nanchang University, Nanchang, Jiangxi Province 330006 People’s Republic of China

**Keywords:** Transcranial direct current stimulation, Disorders of consciousness, Traumatic brain injury, Randomized controlled trial

## Abstract

**Background:**

Disorders of consciousness (DOC) after traumatic brain injury (TBI) raise the mortality of patients, restrict the rehabilitation of patients with TBI, and increase the physical and economic burden that TBI imposes on patients and their families. Thus, treatment to promote early awakening in DOC after TBI is of vital importance. Various treatments have been reported, but there is no advanced evidence base to support them. Transcranial direct current stimulation (tDCS) has shown great potential in promoting neuroelectrochemical effects. This protocol is for a double-blind, randomized, controlled, clinical trial aiming to research the effects and safety of conventional rehabilitation combined with tDCS therapy in patients with DOC after TBI.

**Methods/design:**

Eighty patients with DOC after TBI will be randomized into one of two groups receiving conventional rehabilitation combined with sham tDCS or conventional rehabilitation combined with active tDCS. The intervention period in each of the two groups will last 4 weeks (20 min per day, 6 days per week). Primary outcomes (Glasgow Outcome Scale (GOS)) will be measured at baseline and the end of every week from the first to the fourth week. Secondary outcomes will be measured at baseline and the end of the fourth week. Adverse events and untoward effects will be measured during each treatment.

**Discussion:**

Patients with central nervous system lesions have received tDCS as a painless, non-invasive, easily applied and effective therapy for several decades, and there has been some evidence in recent years showing partial improvement on the level of consciousness of partial patients with DOC. However, reports mainly focus on the patients in a minimally conscious state (MCS), and there is a lack of large-sample clinical trials. This protocol presents an objective design for a randomized controlled trial that aims to study the effectiveness of conventional rehabilitation combined with tDCS therapy for DOC after TBI, to evaluate its safety, and to explore effective and economical therapeutic methods.

**Trial registration:**

Chinese Clinical Trial Registry, ChiCTR1800014808. Registered on 7 February 2018.

**Electronic supplementary material:**

The online version of this article (10.1186/s13063-019-3680-1) contains supplementary material, which is available to authorized users.

## Background

Traumatic brain injury (TBI) is caused by a violent force that affects the head directly or indirectly, and is the leading cause of death and disability among young people around the world [1]. According to epidemiological survey results and expert prediction, by 2020 TBI will become the third largest disease burden in the world. Disorders of consciousness (DOC) refers to the serious impairment of patients’ ability to recognize and perceive the surrounding environment and their own state, which can include coma, vegetative state (VS)/unresponsive wakefulness syndrome (UWS) and a minimally conscious state (MCS) [2]. Many patients with severe TBI will suffer from DOC, and up to 14% of patients with TBI are in a long-term coma or persistent VS after rescue; the longer the duration of coma, the higher the mortality [3, 4]. Therefore, early awakening of patients with TBI can reduce the disability rate and mortality in patients with TBI.

There has been extensive clinical and basic research into treatments for DOC treatments, including electrical nerve stimulation: in DOC this mainly involves median nerve electrical stimulation (MNES) [5–7], spinal cord electrical stimulation (SCS) [8–10], transcranial direct current stimulation (tDCS) [11–13], vagus nerve stimulation (VNS) [14, 15], or deep brain stimulation (DBS) [16, 17]. However, there is no unified DOC treatment guideline [18].

tDCS is a non-invasive brain stimulation technology, which is safe, simple to operate and economical. A previous study [19] has shown that there is no significant untoward effect of tDCS in healthy individuals except for mild tingling (76%), itching (68%), mild burning (54%), and mild pain (25%). tDCS is gradually being applied for treatment of people with motor skills and language impairment after craniocerebral injury, dysphagia, Alzheimer’s disease, Parkinson’s disease, acute and chronic pain, tinnitus, depression and normal subjects, and DOC, as well as in studies such as being tested in healthy individuals [11]. A constant, weak direct current (DC) can effectively pass through the skull and induce bipolar, polarity-related changes through skull conduction on the corresponding cortex. The stimulation effect is determined by the stimulation site, the current intensity, the polarity, and the area of the electrode slice. tDCS works by regulating the activity of spontaneous neuronal networks [12]. At the neuronal level, tDCS regulates excitability in the cerebral cortex. This is mainly due to changes in the polarity of the stimulus, resulting in changes in resting membrane potential depolarization or hyperpolarization, thereby affecting cortical excitability changes in spontaneous neuronal activity. Stimulation usually increases the excitability of the cerebral cortex, and cathodal stimulation reduces the excitability of the cortex [13]. tDCS also increases synaptic plasticity. Besides, some studies have suggested the electrophysiological mechanisms [20–22] and biochemical mechanisms [23, 24] of tDCS, which makes tDCS in DOC more feasible. Nevertheless, clinical research into tDCS combined with conventional rehabilitation in DOC after TBI is rarely reported.

Therefore, the objective of this large-sample, randomized controlled trial is to (1) research the efficacy of conventional rehabilitation combined with tDCS in DOC after TBI; (2) evaluate the safety of tDCS in patients with DOC after TBI; and (3) present the study protocol, the previous results of the clinical trial, ensuring adherence and compliance with the guidelines previously proposed.

## Method/design

### Study design

**Fig. 1:**
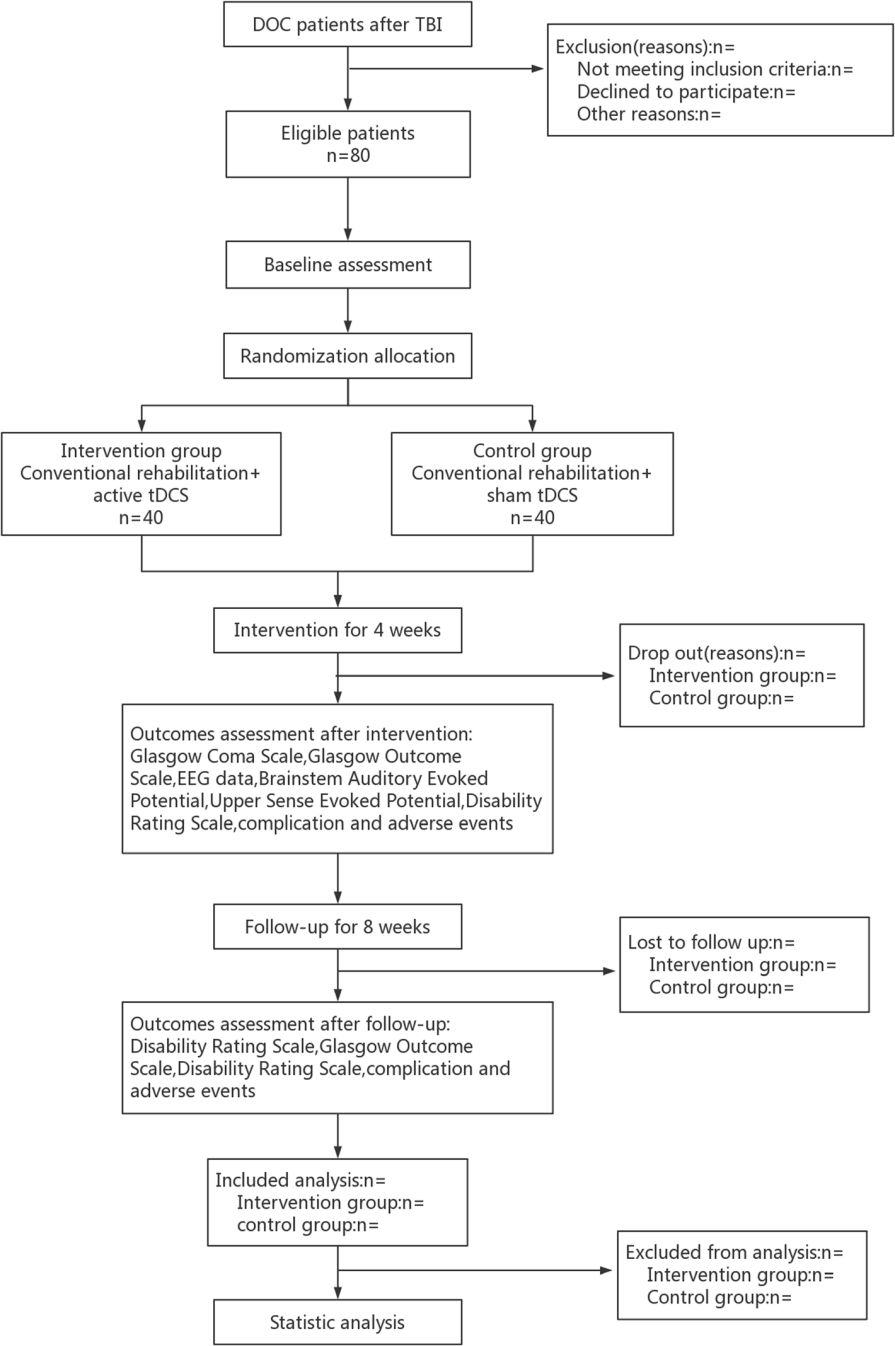
Flow diagram of study design. DOC, disorders of consciousness; TBI, traumatic brain injury; tDCS, transcranial direct current stimulation; EEG, electroencephalogram

The clinical study is a prospective, randomized controlled trial designed with double-blinded assessments, and is being carried out in 80 patients hospitalized with DOC after TBI. All participants will be randomly allocated to the intervention group or control group in a 1:1 ratio. The control group will receive conventional rehabilitation combined with sham tDCS therapy, and the intervention group will receive conventional rehabilitation combined with active tDCS therapy. This trial will comprise a 4-week intervention and 8-week follow-up period. The relative primary outcome (Glasgow Outcome Scale (GOS)) will be measured at baseline and the end of every week from the first to the fourth week. The secondary outcomes (Glasgow Coma Scale (GCS), brainstem auditory evoked potential (BAEP), and upper sense evoked potential (USEP) will be measured and electroencephalogram (EEG)) performed at baseline and the end of the fourth week. During each treatment, adverse events, and untoward effects will be measured. The first affiliated Hospital of Nanchang University (NCU), which will undertake the study, is responsible for training rehabilitation therapists on standard operating procedure and supervising the progress of this trial in all clinical sites. In addition, the randomization and blinding will be performed by an independent statistician from the Center of Evidence Based Medicine, NCU. A flow diagram of this trial is presented in Fig. 1

### Participants and recruitment

The participants will be recruited from the intensive care unit (ICU) and the neurologic intensive care unit (NICU) at the First Affiliated Hospital of Nanchang University. Our study will be advertised on the Internet homepage of First Affiliated Hospital of Nanchang University between December 2019 and December 2020. Posters will select the potential objects who perhaps meet the inclusion criteria by looking through patients’ medical histories via the Computerized Patient Record System (CPRS) and inform patients and their families about this trial is displayed in hospital via phone, or email. Interested families can contact the project leader further. After obtaining informed consent, the prospective participants are screened by a neurologist from the NICU at The First Affiliated Hospital of Nanchang University, according to the inclusion and exclusion criteria. Those who satisfy all criteria are eligible to participate. The eligible individuals are invited to participate in the trial and are directed to a diagnostic assessment by a neurologist, followed by rehabilitation assessments. Consent will be obtained for all study participants from their family members. Data on the design of the study are shown in Table 1.

**Table 1:** Data on the design of the study

Point in time	Screening stage	Treatment period 1 (1 week)	Treatment period 2 (2 weeks)	Treatment period 3 (3 weeks)	Treatment period 4 (4 weeks)
Informed consent	X				
Inclusion and exclusion criteria	X				
Withdrawal, drop out and termination criteria		X	X	X	X
Basic information	X				
Past medical history	X				
Therapeutic parameter record		X	X	X	X
Glasgow Coma Scale (GCS)	X	X	X	X	X
Glasgow Outcome Scale (GOS)		X	X	X	X
EEG	X				X
Brainstem auditory evoked potential (BAEP)	X				X
Upper sense evoked potential (USEP)	X				X
Overall efficacy evaluation					X
Complications	X	X	X	X	X
Adverse events recorded	X	X	X	X	X

*EEG* electroencephalogram

### Inclusion and exclusion criteria for the trial

Patients who meet the following criteria will be deemed eligible for the trial: coma: (1) male or female patients aged 18–65 years; (2) severe traumatic brain injury that leads to coma; (3) coma that lasts more than a week; (4) magnetic resonance imaging (MRI) of the head demonstrates no obvious shift, structural damage, extensive necrosis of the brain structure change and obvious brain stem (not including the pyramidal tract) or thalamic lesions with lesions in each lobe not exceeding 30% of the scope of one side of the brain; (5) stable condition and stable vital signs; and (6) the families of the patients volunteered the patient to participate in the study and provided signed informed consent.

VS/UWS and MCS: (1) male or female patients aged 18–65 years; (2) diagnosis as established by the Multi-Society Task Force in 1994 and the current internationally recognized standards for plant status diagnosis; conforming to the current internationally recognized minimum state diagnostic criteria for international studies, such as reported by Giacino in 2002; (3) the course of illness is more than one month; (4) MRI of the head demonstrates no obvious shift, missing, extensive necrosis of the brain structure change and obvious brain stem (not including pyramidal tract) or thalamic lesions, with lesions in each lobe lesions not exceeding 30% of the scope of one side of the brain; (5) stable condition and stable symptoms; (6) the families of the patients volunteered the patient to participate in the study and provided signed informed consent .

Patients who meet the following criteria will be excluded from the trial: (1) the use of sedative sleeping pills, anesthetics, psychotropic drugs, muscle relaxants or Na^+^ and Ca2^+^ channel blockers (such as carbamazepine, etc.) during the evaluation period; (2) ventilator dependence; (3) the course of illness is longer than 1 year; (4) The presence of any materials within the body that would contraindicate MRI, such as pacemakers, dentures, metal prostheses, etc., or open craniocerebral injury or skull defects that would contraindicate electromagnetic stimulation; (5) history of epileptic seizures or epileptic seizures confirmed clinically by EEG; (6) comorbid serious diseases (such as heart, liver, and kidney failure); (7) progressive neurological diseases such as central nervous system tumor or transsexual disease; (8) fever, electrolyte disturbance or unstable vital signs; (9) pregnancy; (10) local skin injury or inflammation; (11) hemostasis, coagulation, and anticoagulation dysfunction, as well as people on anticoagulants; (12) acute massive cerebral infarction; and (13) hyperalgesia in the stimulus area.

### Sample size

The sample size calculation is based on improvement in scores on the Glasgow coma scale (GCS). According to similar published articles [25], the GCS scores after active tDCS and sham tDCS are respectively (12.44 ± 2.51) and (10.43 ± 1.90), *n* = 38. According to a similar study [26], treatment with conventional rehabilitation combined with tDCS can probably improve the GCS by 2.01 points on average in participants receiving this intervention compared to those in the control group. Improvement will be measured according to the same sample size of the estimation formula:

$$ n=2\left[{\left({u}_a+{u}_{\beta}\right)}^2{\sigma}^2\right]/{\delta}^2 $$

With a type I error of 5% (*α* = 0.05) and 90% power (*β* = 0.10), the estimated required sample size is 33 participants per group. Allowing for a 20% dropout rate during the study, a minimum total of 80 participants is needed to reach the target of 40 participants per group.

### Randomization and allocation concealment

Block randomization will be applied in this study. The participants will be divided into the “coma group”, “MCS group” or “VS group”, depending on their type of consciousness disorder. Each eligible participant will then be randomly assigned to either the intervention group or control group according to the 1:1 equal proportion rule. The sequence of random allocation will be generated using the PLAN function of the statistical software SPSS 24.0 (IBM,USA); this will be undertaken by an independent statistician from the Center of Evidence Based Medicine, NCU, who will not be involved in this trial. Also, the sequence of random allocation will be concealed to outcome assessors and the statistical analyst. The eligible participants’ allocation will also be concealed from their caregivers and from the therapists (including acupuncturist and cognitive therapists) after the participants’ baseline information has been assessed, and the therapists will be arrange the patients’ allocated treatments.

### Blinding

Due to the double-blind implementation of this study, “third party” personnel who are not involved in the experiment will manage and supervise the implementation of the blinding method: (1) the computer-generated random numbers are placed in an airtight envelope. The patients receive the envelope according to the order of their inclusion in the study and are not allowed to open the envelope. The tDCS stimulator mode has been preset as A mode and B mode, and the specific implementer of the project does not know what kind of stimulation AB represents (A is active stimulus, B is sham stimulus); (2) according to the principle of the blinding method, the efficacy of the treatment is evaluated by the third-party evaluators who do not know the grouping. The evaluators do not participate in the recruitment and treatment of patients, and the researchers, operators and statisticians work separately and independently; (3) in order to prevent the subjective tendency of the analyst in the process of data analysis, the first unblinding is performed before the completion of the statistical analysis, that is, the analyst knows that the patients are divided into two groups, but does not know which is the intervention group. After the statistical analysis, the second unblinding is performed to determine which group is the intervention group.

### Ethics issues

The study protocol and consent forms have been approved by the Ethics Committee of the First Affiliated Hospital of Nanchang University (NO: Clinical medicine ethical review [2015] 043). Consent will be obtained from family members of all study participants.

### Baseline tests

Descriptive data will be collected before randomization. The descriptive data include gender, age, height, weight, nationality, education level, employment status, clinical syndrome (left-sided or right-sided brain injury), and the type, degree, and location of the TBI at diagnosis. Vital signs such as blood pressure, heart rate, respiratory rate, and body temperature will also be measured. The GCS and GOS, the tools used to assess the severity of DOC, will be applied at baseline to determine whether the two groups are consistent before the intervention.

### Intervention

The intervention group will receive conventional rehabilitation combined with active tDCS therapy, while the control group will receive conventional rehabilitation combined with sham tDCS therapy. The conventional rehabilitation consists of hyperbaric oxygen, cerebellar nuclear stimulation, limb electrical stimulation, and passive limb range-of-motion training. The treatments will be performed six times per week for 4 weeks. For quality management, all procedures performed by the therapists participating in this study will be standardized, including the study protocol, treatment methods, and assessments.

### Basic treatments

All participant will receive basic treatments following the *Guidelines for the Management of Severe Traumatic Brain Injury, Fourth Edition* [27]. Based on this guideline and the patient’s condition, the treating physician will manage each patient, including management of the use of drugs and prevention of complications.

### Hyperbaric oxygen

All participants will receive hyperbaric oxygen therapy. The hyperbaric oxygen chamber model is the *YC3200/0.3-22VII*. According to a similar study [28], the treatment parameters are as follows:

(1) treatment of pressure is 1.8~2.0 atmospheres absolute (ATA) ; (2) plus and minus pressure time is 25 min; (3) after adjusting the pressure via the voltage regulator, wearing a mask to absorb pure oxygen 30 min for twice and breathe in cabin air for 10 min during the interval of twice absorbing pure oxygen; or breathe pure oxygen 20 min for thrice and breathe in cabin air for 5 min during the interval of thrice absorbing pure oxygen.

### Cerebellar nuclear stimulation

All the participants will receive cerebellar nuclear stimulation. The model of the electro-bionic stimulator is the NK - IA05. A crescent electrode will be applied behind the ear to improve the posterior circulation in the brain. According to normal tolerance, the current intensity is about 15 mA, and the stimulus time is 30 min per treatment.

### Limb electrical stimulation

All the participants will receive limb electrical stimulation. This will be performed with 4 cm × 4 cm square electrodes, which will be attached to the extensor carpi radialis and the abdominal muscles of the tibialis anterior. The current intensity is according to use in sport, and the stimulus time is 30 min per treatment.

### Passive limb range-of-motion training

All the participants will receive passive limb range-of-motion training. The therapist will peform a total range of joint activities at the shoulder, elbow, hip, and knee. The training lasts 30 min per treatment.

### Active tDCS

The interventional group participants will receive active tDCS therapy. The anode of the electrode will be located in the dorsolateral prefrontal cortex (DLPFC) at the left side of the forehead, and the cathode will be located in the superior margin of the right orbit. The current intensity is 1–2 mA, and the square electrode has a range of 5 cm × 7 cm. The treatment parameters are as follows: (1) 20 min per treatment; (2) once per day; (3) six times per week.

### Sham tDCS

The control-group participants will receive sham tDCS therapy. The anode of the electrode will be located in the DLPFC at the left side of the forehead, and the cathode will be located in the superior margin of the right orbit. The current is only input in each 15 s of the initial stage, and there is no current output in the middle 19.5 min of the sham therapy. The remaining parameters are consistent with the active stimulus.

### Study endpoints

The study endpoints are as follows:

1. If serious adverse reactions occur during the test, the test will be terminated in time to protect the subjects
2. In the case of serious complications or deterioration of the condition of the subject in the test, the test will be terminated if emergency measures are required
3. If the subject is required to withdraw from the clinical study, the test will be terminated
4. If the patient is not cooperative and does not obey the treatment, and the treatment staff repeatedly explains that the treatment is ineffective, the test will be suspended

The investigator will record in detail the reason and time of a participants’s withdrawal from the study.

### Follow up

After the 4-week treatment period, patients will be followed up for 8 weeks. The medical staff will follow up the participants via telephone. Each participant is contacted once every 2 weeks, keeping record of the medications and rehabilitation therapies. On the final week of the follow-up period (8 weeks post intervention), participants will be referred for clinical evaluation in order to assess their prognosis and disability condition.

### Outcome measures

In this study, primary outcomes will be measured at baseline and the end of every week from the 1st to the 4th week. Secondary outcomes will be measured at baseline and the end of the 4th week. During each treatment, adverse events and untoward effects will be assessed. After follow up (8 weeks), the prognosis and disability condition outcomes (GOS and Disability Rating Scale (DRS)) will also be evaluated. All outcome assessments will be independently performed by experienced and blinded assessors, the same one professional rehabilitation physician. A summary of all the measures in the trial is shown in Table 1.

### Primary outcomes

#### Glasgow Outcome Scale

The GOS is used to assess the recovery and outcome of patients with TBI. The severity of disability is divided into 5 levels according to indicators such as whether the patients can recover to undertake work, study, and self-care. The 5 levels are death, vegetative state, severe disability, moderate disability and good recovery.

### Secondary outcomes

#### Glasgow Coma Scale

The GCS was first introduced by Jones et al. in 1979 [29]. It is a brief scale with 15 points in total, divided into 3 items: Eye Opening Response (E), Verbal Response (V) and Motion Response (M).When the resulting GCS is between 13 and 15 points, and the duration time of coma after injury is shorter than 20 min, this means that the patient has light TBI; when the resulting GCS is between 9 and 12 points, and the duration time of coma after injury is between 20 min and 6 h, this means that the patient has moderate TBI; when the resulting GCS is between 6 and 8 points, and the duration time of coma after injury is longer 6 h, this means that the patient has severe TBI; and when the resulting GCS is between 3 and 5 points, this means that the patient has extremely severe TBI.

### Brainstem auditory evoked potential

The BAEP is sensitive in detecting brain stem auditory pathway lesions, and has been used for more than two decades. Many scholars use it to assess brain function in acute critical illness, and it has been widely used to evaluate the degree and prognosis of TBI [30, 31]. Greenberg first proposed the BAEP grading standard in 1977 [32]. Later, many scholars took this as the standard for clinical research and improvement; other new classification methods have also been proposed. At present, there are more than ten kinds of BAEP grading standards. The Hall grading standard [33] is one of the most detailed. It divides the abnormal BAEP condition into 4 levels: level 1 normal and level 2 mildly abnormal: I ~ V wave well-differentiated, but changeful in any of the following situations such as I, III, or (and) V wave PL extend, I ~ III,III ~ V or (and) I ~ V wave PL extend, III ~ V/I ~ III peak to peak latency phase ratio > 1, V/I amplitude ratio < 0.5; level 3: moderately abnormal: III and (or) V wavelength division poor, poor repeatability, or missing V wave; and level 4: severely abnormal: only the I-wave or both waves are absent.

### Upper sense evoked potential

The USEP is used to stimulate the upper skin or peripheral nerve. Firstly, the nerve impulses transmit to the spinal cord along the afferent neural pathway. Secondly, the nerve impulses transmit to the thalamus along the ascending sensory pathway of the spinal cord. Thirdly, the nerve impulses transmit to the cerebral cortex central posterior sensory area along thalamic central radiation. Finally, the corresponding contra hemisphere cortex of the stimulated upper skin can be activated [34]. Amantini [35] suggests that UESP has high accuracy in predicting the prognosis in DOC.

### EEG data

The EEG is an effective method to evaluate and predict brain function [36]. We will measure changes in participants’ brain function.

### Safety assessments

The medical staff will record adverse events (AEs) that occur at any time during treatment. When a serious AE occurs, the Ethics Committee of the First Affiliated Hospital of Nanchang University will determine whether the participant needs to be withdrawn from the study. We will treat AEs free of charge.

### Data collection and management

General baseline information will be collected about patients when they are sent to the hospital and will include the following: age, sex, nationality, education level, disease type, course of illness. Major indicators are assessed at the end of the 1st, 2nd, 3^rd^, and 4th week of treatment, including application of the GOS after 4 weeks of treatment, all secondary index evaluations: the BAEP, body feeling on USEP, and EEG data. For each treatment, safety is assessed in terms of adverse events and adverse reactions.

Outcomes will be assessed using the GOS at the end of 1st, 2nd, 3rd, and 4th week, and will be performed using the BAEP, USEP, and EEG at the end of the 4th week of treatment. Complications during follow up will also be assessed. If the patient is still in the hospital, the investigator may visit the patient on the ward to go through the evaluation. If the patient has been discharged, his or her legal representative will be told to come back in due time for assessment after leaving the hospital. If the patient does not arrive, the investigator will try to contact the patient or the family members by telephone. Other possible methods may also be used to explain the situation and complete the outcome assessment. If all attempts fail, no further contact will be made, and the patient will be recorded as lost to follow up.

All the data will be collected by independent investigators who are blind to the patient’s allocation. Each local study center will assign a specific investigator at the beginning of the trial. This investigator will be excluded throughout treatment of all the participants unless asked by clinicians to perform the assessment.

All variables specified in the protocol will be documented on standardized hard-copy case report forms (CRFs) in all participating centers. When the 8-week follow up is complete, data in the CRF of each patient will be validated for completeness, consistency, and plausibility, by an independent investigating physician in a local center. Then the CRF will be transmitted to the Center of Evidence Based Medicine, NCU, which will be responsible for the development of a central database, and data entry and storage. At the end of the trial, the database will be locked and sent to the study statistician for analysis based on a predetermined statistical analysis plan.

#### Data Safety and Monitoring Committee (DSMC)

The project will be monitored by the DSMC, initiated by the clinical trial center of The First Affiliated Hospital of Nanchang University, and composed of specialists in rehabilitation, ethics, statistics, and methodology. The DSMC will audit the study through regular interviews or telephone calls.

### Statistics methods

#### Statistical analysis

SPSS21.0 software will be used to analyze the test results. In the descriptive analysis of the sample, continuous variables will be expressed by mean and standard deviation for normally distributed data, and median and interquartile range for non-normally distributed data. Normally distributed data variables will be statistically compared between groups using the *t* test. Ordinal-level variables with a non-normal distribution will be statistically compared between groups using the Mann–Whitney *U* test. Measures with a discrete distribution will be expressed as percentages and analyzed using the chi-squared (χ2) or Fisher’s exact test as appropriate. A general linear model or logistic regression model will be applied to adjust for confounding influences if necessary.

We will compare baseline characteristics between groups using the *t* test or Mann–Whitney test for continuous variables and the Pearson chi-squared or Fisher’s exact test for categorical variables. If statistical significance appears, the inequality factors will be treated as confounding variables in the final efficacy analysis. For comparison of the primary or secondary outcomes between groups, the *t* test or non-parametric tests will be used to analyze continuous data, and the Pearson chi-squared or Fisher exact test to analyze categorical data. To control for possible confounding variables, linear models or linear regression will be applied for dependent continuous variables and logistic regression models for dependent categorical variables. Subgroup analysis of the primary outcomes will be stratified by participants’ sex. Analysis of variance will be used for the repeated measurement data. Analysis of the primary and secondary outcomes will be on an intention-to-treat (ITT) and a per-protocol (PP) basis. The result of the ITT analysis will be compared with that of the PP analysis to determine whether the results are consistent. Missing data will be handled according to the method of last observation carried forward. Adverse events will be listed and analyzed using the chi-square test or Fisher’s exact test.

## Discussion

As a new method in brain function research and treatment, tDCS is painless, noninvasive, easy to apply, and effective. At present, it has been studied and applied in the fields of depressive brain dysfunction [36], Parkinson’s brain dysfunction [37], epilepsy brain dysfunction [38–41], post-stroke brain dysfunction [42, 43], addiction brain dysfunction [44, 45] etc., but has rarely been used in the study of patients with consciousness disorder after TBI.

Studies have shown that transcranial electrical stimulation may regulate brain function by activating the corresponding cerebral cortex through the electrical current [46] and changing the concentration of certain chemicals [47]. In addition, a study also found that tDCS causes a significant increase in cerebral blood volume (CBV) and cerebral blood flow (CBF) and a decrease in the average mean transit time (MTT), which suggests better oxygen delivery to the tissues [48]. This may be one of the physiological ways in which tDCS improves disturbances in consciousness.

tDCS regulates the synaptic microenvironment, such as by altering the mediation of N-methyl-D-aspartate receptor at synaptic level on long-term potentiation and long-term inhibition processes. Studies have also shown that the involvement of tDCS at the synaptic level is also involved in the modification of gamma-aminobutyric acid and dopaminergic and other protein systems [49]. There is also an immediate effect in tDCS, and studies have shown that tDCS can stimulate changes in regional cerebral blood flow. Anodic stimulation of tDCS can induce the increase of cerebral blood flow in the related areas, and the increase may be up to about 17.1%. When the stimulation stops, the blood flow returns to baseline level. When the cathodic stimulation stops, the blood flow increases by about 5.6%. When the stimulation stops, the blood flow decreases by about 6.5% compared with the baseline, and it lasts to the later stage of stimulation. A study [50] on functional MRI and positron emission scanning found that tDCS in the M1 region could lead to the intensification of other regions besides the M1 region, and tDCS could also affect the activity of neurons in thalamus region. Thus, although tDCS acts in the cerebral cortex, it is likely to affect other brain tissues through the interconnection of neurons. It has also been shown that tDCS stimulation of the F3 position using the EEG 10/20 system can increase the blood flow perfusion in the DLPFC and decrease the function of bilateral thalamic blood flow, suggesting that tDCS may be involved in regulating the functional connection between the DLPFC and the thalamus. Thibaut et al. [51] found that when tDCS is used to stimulate the dorsolateral left prefrontal lobe, the left DLPFC, thalamus and anterior wedge lobe increased in the tDCS-positive subjects. This may be a mechanism to improve the awareness of tDCS disturbances.

The double-blind test design was adopted to ensure the authenticity of the test results. In addition, the GCS, GOS, EEG, BAEP, and USEP were selected as evaluation indexes using the scale score and electrophysiological measurement, so as to comprehensively and objectively evaluate change in the patient’s consciousness disorder. DOC after brain injury makes the prognosis worse and brings a great burden to the family and society. The purpose of this protocol is to investigate the effects of tDCS in patients with DOC after brain injury and to find a new intervention to improve this condition. Finally, as this trial is based on a small sample, it would be of great value to conduct a meta-analysis of the results from other similar studies. Thus, it will provide new medical evidence on the use of tDCS in patients with DOC after TBI, which is one aspect of our future research work.

### Trial status

This protocol version number is V2.1(2019082201). Participant enrollment will start in December 2019. The trial is expected to be completed by the end of December 2020.

## Additional file

Additional file 1: Checklist: Recommended items to address in a clinical trial protocol and related documents*. (DOC 125 kb)

## Data Availability

All data will be archived in an electronic CRF under the responsibility of the First Affiliated Hospital of Nanchang University. The data set generated and analyzed during the current study will be available from the corresponding author on reasonable request.

## Abbreviations

AE: Adverse event
BAEP: Brainstem auditory evoked potential
CRF: Case report form
DBS: Deep brain stimulation
DLPFC: Dorsolateral prefrontal cortex
DOC: Disorders of consciousness
DRS: Disability Rating Scale
EEG: Electroencephalogram
GCS: Glasgow Coma Scale
GOS: The Glasgow Outcome Scale
ICU: Intensive care unit
ITT: Intention to treat
MCS: Minimally conscious state
MNES: Median nerve electrical stimulation
MRI: Magnetic resonance imaging
NICU: Neurologic intensive care unit
PP: Per protocol
TBI: Traumatic brain injury
tDCS: Transcranial direct current stimulation
USEP: Upper sense evoked potential
UWS: Unresponsive wakefulness syndrome
VNS: Vagus nerve stimulation
VS: Vegetative state
